# Renal Artery Stenosis Precipitates Hyponatremic Hypertensive Syndrome and Posterior Reversible Leukoencephalopathy

**DOI:** 10.3389/fped.2015.00040

**Published:** 2015-05-07

**Authors:** Pranav Parikh, Danielle Duhame, Laura Monahan, Robert Woroniecki

**Affiliations:** ^1^Department of Pediatrics, Stony Brook University School of Medicine, Stony Brook, NY, USA

**Keywords:** renal artery stenosis, hypertension, posterior reversible leukoencephalopathy, hyponatremia, seizure, revascularization

## Abstract

**Background:**

Hyponatremic hypertensive syndrome (HHS) is an uncommon disorder usually encountered in the adult population with unilateral renal artery stenosis and is under-recognized in the pediatric population.

**Case diagnosis/treatment:**

A 19-month-old male presented with new-onset status epilepticus associated with neurological sequelae, and hypertension to a high of 248/150 mmHg. Lab work revealed significant hyponatremia, elevated peripheral renin activity, and increase in aldosterone and ADH levels. A diagnosis of HHS was made. Initial analysis revealed a high-grade proximal renal artery stenosis by magnetic resonance imaging (MRI) and angiogram. Electroencephalogram and an MRI of the brain demonstrated characteristic abnormalities of the left temporal–parietal regions consistent with posterior reversible leukoencephalopathy syndrome (PRES). The patient responded to right renal artery balloon dilation and stent placement. Since intervention and close blood pressure control with Amlodipine, the patient has been free of seizures and is neurologically intact.

**Conclusion:**

We report a case of malignant hypertension in a 19-month-old male secondary to renal artery stenosis with associated HHS and PRES. Prognosis of PRES in children with renal disease is excellent. Prompt intervention may offer near complete resolution of physiologic and symptomatic effects of HHS and PRES due to high-grade renal artery stenosis. This report was written with parental consent for de-identified case presentation and radiographs for the educational benefit of other medical professionals.

## Introduction

Renovascular disease is responsible for approximately 5–25% of all childhood hypertension (1–4). Fibromuscular dysplasia of the renal arteries predominates in this setting, representing 60% of cases (3). Hyponatremic hypertensive syndrome (HHS) is of special concern in patients with both hyponatremia and renovascular hypertension. HHS has been typically reported in the elderly, asthenic females with underlying renal artery atherosclerosis, but is underreported in the pediatric population (5, 6).

Renal artery stenosis may result in critical renal ischemia, leading to renin hypersecretion. Elevated downstream modulators such as angiotensin II and aldosterone potentiate hemodynamic changes, resulting in hypertension and pressure natriuresis. Malignant hypertension on presentation is well reported in adults with HHS, though reports in children are slowly emerging (7–9). We report a 19-month-old male presenting with malignant hypertension and status epilepticus complicated by posterior reversible leukoencephalopathy syndrome (PRES), a recently described disorder incorporating both clinical and radiographic features (10).

## Case Report

### Patient presentation

A 19-month-old African American male was transferred from an outside hospital to our pediatric unit for new-onset status epilepticus and hyponatremia (serum sodium of 126 mmol/L). He initially presented with generalized tonic–clonic seizures unresponsive to rectal Diazepam, but eventually resolved with intravenous Lorazepam after approximately 15 min. Upon admission, the patient appeared lethargic but responsive. Vital signs on admission: blood pressure 218/144, heart rate of 210, respiratory rate was 32, and temperature was 36.8°C. Lab work revealed hyponatremia, an elevated peripheral renin activity, aldosterone and ADH levels, and a normal serum creatinine (Table 1). His weight was 10.6 kg (29th‰ for age) and his height was 87.3 cm (88th‰ for age). His physical exam was unremarkable. He was awake and alert, normal heart, and lung sounds. Abdomen was soft, non-tender, and non-distended with no evidence of organomegaly. Extremities were warm with normal pulses and capillary refill.

**Table 1:** **Review of serum and urine chemistries**.

Chemistries	Serum	Reference values	Spot urine	Reference values
Osmolality	273	280–295 mOsm/Kg	329^a^	500–800 mOsm/Kg
Sodium	128^a^	135–148 mmol/L	18	>20 mmol/L
Potassium	3.2	3.5–5.3 mmol/L	38.5	10–60 mmol/L
Chloride	87	98–108 mmol/L	22	mmol/L
Bicarbonate	24	21–31 mmol/L		
Glucose	89	70–99 mg/dL		
BUN	14	5–20 mg/dL		
Creatinine	0.35	0.50–1.20 mg/dL		
Calcium	9.9	8.6–10.2 mg/dL		
Phosphorus	5.9	4.5–6.7 mg/dL		
Magnesium	2.3	1.6–2.6 mg/dL		
Aldosterone	743^a^	7–93 ng/dL		
ADH	64.6^a^	<0.69 ng/dL		
Peripheral renin activity	137^a^	3–11 ng/ml/min

*^a^Abnormal lab values*.

Electrocardiogram (ECG) showed sinus tachycardia and a waveform characteristic concerning for left ventricular hypertrophy. Echocardiogram demonstrated normal biventricular size with qualitatively mild concentric left ventricular hypertrophy.

Upon review of systems, the family revealed a 1 kg weight loss over the course of 10 days. He was seen by a pediatric gastroenterologist for daily non-bloody, non-bilious emesis, and was treated for gastroesophageal reflux disease (GERD). During this time, he also demonstrated signs of polyuria and polydipsia with water intake increasing to 8–10 bottle per day.

Shortly after admission, his blood pressure increased to 248/150 mmHg. He became lethargic and suffered a second episode of seizure activity, which was successfully treated with Lorazepam followed by a loading dose of Phenobarbital. His blood pressure was titrated with a nicardipine infusion for a target range of 120–140 mmHg systolic and 70–80 mmHg diastolic.

### Initial diagnosis

A renal ultrasound revealed a smaller right kidney (length 6.4 cm) as compared to the left (length 7.6 cm). The right kidney when compared to the left had less cortical thickness and increased echogenicity suggestive of vascular insult. The vessels were not completely visualized via color Doppler.

**Figure 1:**
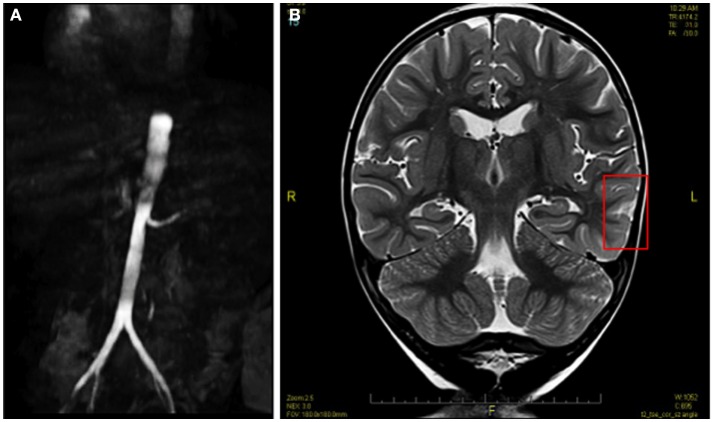
**Pre-angioplasty MRI image showing high-grade proximal renal artery stenosis (A) and hyperintense lesions of PRES (B)**.

**Figure 2:**
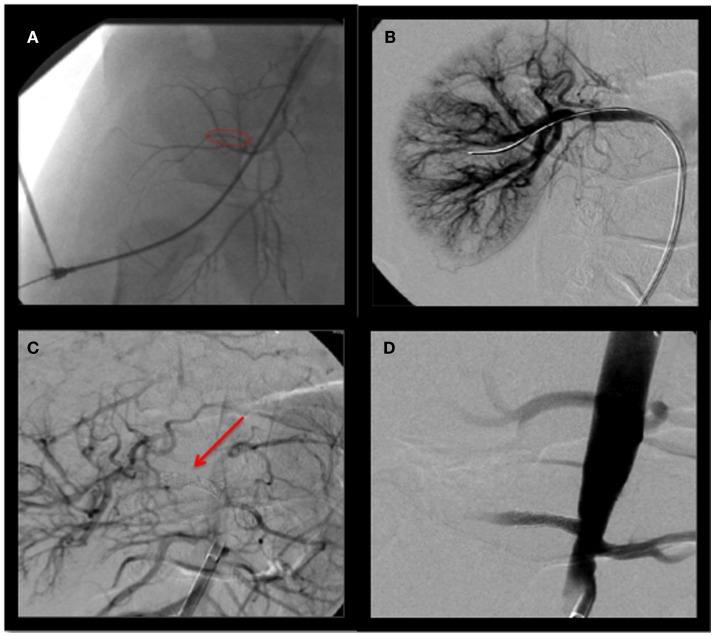
**Angiography of right renal artery (A), pre-balloon dilation and stenting (B), balloon dilation (C) stenting (D), post-balloon dilation and stenting**.

MRI of the brain without contrast demonstrated features consistent with PRES (Figure 1). MRI of the abdomen with contrast showed a small right kidney and a high-grade proximal renal artery stenosis (Figures 1 and 2A). Upper pole hypointense lesions of the right kidney consistent with long-standing ischemic insults. Diagnostic angiography later confirmed a complete occlusion of his renal artery accompanied by a 20% narrowing of the perirenal aorta. Video EEG was performed which exhibited frequent, focal spikes and sharp waves in the left temporal region. Seizure activity was thought to emanate from accrued pathological alterations in the brain regions specified.

### Outcome

The right renal artery was balloon dilated to 50% as shown in Figure 2B. This was complicated by renal artery re-stenosis, with a peak systolic velocity of 200 cm/s (normal <180 cm/s) on serial renal Doppler ultrasounds. Repeat angiogram showed a 30% opening. Following repeat balloon angioplasty, the patient’s renal artery progressively re-occluded over 15 min requiring a 4 × 14 Genesis stent to maintain adequate flow, as shown in Figures 2C,D. He was weaned off the nicardipine to amlodipine 2.5 mg in a.m. and 5 mg in p.m. to good effect. Since his hospitalization, the patient has been free of seizures and is neurologically intact.

## Discussion

HTN in children is more likely due to a secondary cause if the BP is elevated to greater than the age-sex-height specific 99th ‰ + 5 mmHg (Stage 2 HTN range) or if the child is young at presentation. Renal etiologies explain many secondary causes, with renovascular hypertension one of the most common renal forms of secondary HTN. Renovascular hypertension should be suspected in children with suspected secondary HTN, as well as in those with raised peripheral plasma renin, moderate hypokalemia, or HTN so severe that it requires more than two agents to normalize the BP (4). A number of etiologies of renovascular disease have been described in the literature and are displayed in Table 2. HTN and hyponatremia in the setting of renovascular disease is better known as HHS.

**Table 2:** **Causes of renovascular hypertension in the pediatric population**.

Categories	Specific etiologies
Anatomical	Fibromuscular dysplasia, extrinsic compression
Vasculitis	Kawasaki disease, polyarteritis nodosa, Takayasu’s disease
Syndromes	Neurofibromatosis 1, tuberous sclerosis, Marfan’s syndrome, William’s syndrome
Localized tissue damage	Trauma, radiation, umbilical artery catheterization
Congenital	Congenital rubella

Presenting symptoms of HHS include central nervous system (CNS) abnormalities such as headache, confusion, and most importantly seizures as in this case, along with other neuro-behavioral complaints, weight loss, polydipsia, and polyuria (11, 12). Laboratory abnormalities include hyponatremia, hyperreninemia, hypokalemia, hyperaldosteronemia, hypocholremic alkalosis, and elevated urine sodium, and protein levels. The mechanism of hyponatremia in HHS is thought to be due to both diuresis and natriuresis as a response to hypertension and increased levels in ADH, resulting in water conservation as a response to volume depletion. Severe hyponatremia can lead to brain edema and subsequent seizure activity.

Posterior reversible leukoencephalopathy syndrome is underreported in children and thus far has only been described in cases in which the primary diagnoses are glomerulonephritis, systemic lupus erythematosus, Henoch–Schönlein purpura/nephritis, hemolytic uremic syndrome, or hematologic/oncologic diagnoses (10, 13, 14). Risk factors identified for development of PRES include HTN, infection, and presence of pro-inflammatory cytokines, collagen vascular disease, organ transplant, and immunosuppressive drugs (14, 15).

The pathophysiology of PRES secondary to HTN is thought to be due to reduced cerebral blood flow and capillary leakage with endothelial dysfunction leading to hypoperfusion and ischemia. The posterior region of the brain is more susceptible to ischemic pathology due to its impaired autoregulation and decreased sympathetic innervation. HTN, however, is not present in 20–40% of cases and the pathophysiology of other etiologies is not well understood (15). Early diagnosis is important for initiation of treatment to prevent permanent sequelae. Clinical features at presentation include hypertension, seizures (42%), visual changes (33%), headache (17%), and altered mental status (AMS) (8%) (10). The diagnosis of PRES is best confirmed with MRI with FLAIR or fluid-attenuated inversion recovery, which makes white matter lesions and edema appear bright, where bilateral white matter abnormalities in watershed areas of posterior regions and vasogenic subcortical edema are expected (14, 15).

The mainstay of treatment for HHS and PRES in the setting of renovascular disease is to first decrease the blood pressure while replacing fluid losses. Management ultimately depends on the underlying cause of hypertensive disease; in this case, renovascular disease (11). Initial antihypertensive therapy most commonly includes calcium channel blockers or beta blockers. Ace-inhibitors are generally contraindicated as first line anti-hypertensives. In cases of bilateral renal artery stenosis, or cases with solitary kidney, they can result in dilation of glomerular efferent arteriole, decreased glomerular filtration rate (GFR), and acute kidney injury (4). Most children eventually need to undergo revascularization with endoscopic or surgical intervention. Indications for more immediate intervention include uncontrolled HTN, evidence of end organ damage, and renal failure.

Studies have shown high success rates of revascularization procedures in patients with fibromuscular dysplasia or mid-aortic syndrome in comparison to those who have diffuse abnormalities of small infrarenal arteries (16). However, revascularization procedures have varied success rates, depending on the extent of RVD, with HTN being cured in 28–94% of cases with percutaenous transluminal renal angioplasty (PTRA) and in 36–70% with revascularization surgery (4). Restenosis, however, is not uncommon. Extensive dissection of our patient’s renal artery during balloon dilation may have contributed to an initial failure. We believe further research exploring the pathophysiological factors involved in re-stenosis may add to the care of these patients. Nevertheless, a complete cure rate of 27.3%, improvement rate of 45.5%, and restenosis rate of 40.9% over a median post-operative interval of 11.8 months has been shown in children who have undergone PTRA for renovascular disease (17). Nephrectomy may be required if PTRA fails or if affected kidney contributes to <10% of renal function (11).

## Concluding Remarks

The prognosis of PRES in children with renal disease, such as our patient, is excellent and most patients recover within a few weeks with minimal neurological sequelae (14). In this case, revascularization with angioplasty, and subsequent stent placement resulted in the resolution of this patient’s HHS and PRES.

## Conflict of Interest Statement

The authors declare that the research was conducted in the absence of any commercial or financial relationships that could be construed as a potential conflict of interest.
